# Exploring the priorities of ageing populations in Pakistan, comparing views of older people in Karachi City and Thatta

**DOI:** 10.1371/journal.pone.0304474

**Published:** 2024-07-05

**Authors:** Bilal Ahmed Usmani, Maryam Pyar Ali Lakhdir, Sonia Sameen, Saila Batool, Maria Lisa Odland, Dina Goodman-Palmer, Sandra Agyapong-Badu, Lisa R. Hirschhorn, Carolyn Greig, Justine Davies

**Affiliations:** 1 Department of Community Health Sciences, Aga Khan University, Karachi, Pakistan; 2 Department of Biomedical Engineering, NED University of Engineering and Technology, Karachi, Pakistan; 3 Dalla Lana School of Public Health, University of Toronto, Toronto, Canada; 4 BSc Medical Bioscience Monash University Malaysia, School of Science, Subang Jaya, Malaysia; 5 Institute of Applied Health Research, University of Birmingham, Birmingham, United Kingdom; 6 Department of Obstetrics and Gynecology, St Olav’s Hospital, Trondheim University Hospital, Trondheim, Norway; 7 Institute of Life Course and Medical Sciences, University of Liverpool, Liverpool, United Kingdom; 8 Malawi-Liverpool-Wellcome Trust Research Institute, Blantyre, Malawi; 9 Department of Public Health and Nursing, Norwegian University of Science and Technology, Trondheim, Norway; 10 School of Sport, Exercise and Rehabilitation Sciences, University of Birmingham, Birmingham, United Kingdom; 11 Department of Medical Social Sciences and Havey Institute of Global Health, Feinberg School of Medicine, Northwestern University, Evanston, IL, United States of America; 12 NIHR Birmingham Biomedical Research Centre, University Hospitals Birmingham NHS Foundation Trust and University of Birmingham, Birmingham, United Kingdom; 13 MRC-Versus Arthritis Centre for Musculoskeletal Ageing Research, University of Birmingham, Birmingham, United Kingdom; 14 Department of Global Health, Centre for Global Surgery, Stellenbosch University, Cape Town, South Africa; The Aga Khan University, PAKISTAN

## Abstract

As a lower middle-income country, Pakistan faces multiple issues that influence the course of healthy ageing. Although there is some understanding of these issues and the objective health outcomes of people in Pakistan, there is less knowledge on the perceptions, experiences, and priorities of the ageing population and their caretakers (hereafter, “stakeholders”). The aim of the study was to identify the needs and priorities of older adults and stakeholders across both urban and rural locations. We sought to explore the views of two groups of people, older adults and stakeholders on topics including the definition of ageing as well as areas of importance, services available, and barriers to older people living well. Two-day workshops were conducted in one rural city, Thatta and one urban city, Karachi. The workshops were designed using the Nominal Group Technique, which included plenary and roundtable discussions. The responses were ranked through rounds of voting and a consensus priority list was obtained for each topic and group. Responses were categorized using the socio-ecological framework. Responses were compared between stakeholders and older people and between different geographical areas. 24 urban and 26 rural individuals aged over 60 years and 24 urban and 26 rural stakeholders attended the workshops. There were few areas of agreement with respect to both geographical region and participant group. Comparing older adults’ definition of ageing, there was no overlap between the top five ranked responses across urban and rural locations. With respect to areas of importance, there was agreement on free health care as well as financial support. In terms of barriers to healthy ageing, only nation-wide inflation was ranked highly by both groups. In addition, there were relatively few areas of congruence between stakeholder and older adult responses, irrespective of location, although engagement with family, adequate nutrition and monetary benefits were responses ranked by both groups as important for healthy ageing. Both groups ranked issues with the pension system and financial difficulties as barriers. When categorized using the socio-ecological model, across all questions, societal factors were prioritized most frequently (32 responses), followed by individual (27), relationship (15), and environment (14). Overcoming barriers to facilitate healthy ageing will require a multi-faceted approach and must incorporate the priorities of older individuals. Our results may serve as a guide for researchers and policymakers for future engagement and to plan interventions for improving the health of the ageing population in Pakistan.

## Introduction

The Islamic Republic of Pakistan (hereafter “Pakistan”), is a country in South Asia, ranked 150^th^ out of 189 countries on the latest United Nations Human Development Index [1,2]. As per 2020 statistics, it is the world’s fifth-most populous country, with a population reaching 222,903,998 with a growth rate of 2.10% [3]. Currently, 6% of Pakistan’s population (7.3 million people) is over the age of 60 years, and 40% of households include an older individual [4]. The proportion of older people living in Pakistan is estimated to double by 2050 to 42.8 million older adults, accounting for 12.4% of the population and rising at a much faster rate than other population age groups [4–8]. In 2019, older people constituted 6.77% of the total population in rural areas and 6.38% in urban areas [1]. Pakistan’s average life expectancy has extended rapidly to reach 67.7 years (male: 65.8 years/female: 69.8 years) and is estimated to reach 76.7 years by 2050 [9].

Unfortunately, in Pakistan, most national policies focus on younger populations; older individuals are largely missing from national initiatives and programs [8]. A survey study by the Pakistan National Centre for Ageing (PNCA) in 2010, shows that 47% of older individuals do not have the capacity to pay for health care, safe shelter, food, and clean water [10]. Frailty, lack of knowledge of self-protection of rights, and poverty further contribute to poor quality of life and thus hinder active ageing [11–13]. Combined with a lack of resources, the extended family system is declining, with people moving towards the nuclear family structure [14]. This transition has resulted in lower numbers of older individuals residing at home with their families, with negative impacts on the older individuals’ involvement in decision-making, physical and emotional wellbeing, and social standing [5,15].

However, despite evidence exploring the circumstances of older people in Pakistan, there has been little research that has explored the subjective needs and priorities of older people and the stakeholders who look after them (family members caring for their elders as well as local caretakers hired to support an older individual). More research is needed to determine where to invest scarce resources to plan services which meet the explicit needs of older people. Additionally, contrasting the priorities of stakeholders with those of older people is likely to show whether stakeholders, who may have greater voice to influence change, understand and can articulate the needs defined by older people.

The World Health Organization defines healthy ageing as “a process of maintaining functional ability to enable wellbeing in older age” [16]. The aim of this study was to assess the needs and priorities for healthy ageing as expressed by older people and relevant stakeholders in rural and urban areas of Pakistan. A secondary aim was to characterize the mental wellbeing of the older people participating in the workshop.

## Methods

### Study setting

The cross-sectional study took place in two socioeconomically different areas of Pakistan’s Sindh province; Karachi and Thatta. Karachi, chosen as the urban location for the study, is the capital of Sindh province, and the largest and most populous city in Pakistan, with an estimate of 16.1 million (16,093,786) inhabitants (2020). Contrastingly Thatta is the capital of the district Thatta; locally called Laar, located in the southern-most area of Sindh, with a total population of approximately 1 million. According to the UNDP Human Development Index report for Pakistan (2017), there exist significant socio-economic disparities between the two chosen regions. According to the report Karachi city lies in the ‘High Human Development’ category while Thatta falls in the ‘Low Human Development’ Index category [17]. The Centre for Innovation in Medical Education (CIME) within the Aga Khan University campus, located in the central part of the city, was chosen for all the workshop sessions conducted in Karachi’s urban area. Makli Gymkhana in Thatta, a family-style restaurant, with large hall and meeting rooms, near village settlements, was chosen as the workshop site in the rural area.

### Participants

#### Older adults

The inclusion criteria for the older participants recruited in both the rural and urban areas were that they must be Pakistani individuals who spent a maximum duration of their lifetime in Pakistan, were of ages 60 years and above and for whom the workshop location was accessible. These participants were identified through a community mobilizer and a study coordinator and selected purposively to ensure that they broadly represented the age and socioeconomic variations across each study area. Apart from those older individuals who were not Pakistani, and unable to travel to the workshop due to severe disability, those who could not provide informed consent to take part in the workshop sessions were excluded from the study.

#### Stakeholders

Caregivers, such as members of senior councils, Ministry of Local Government, Ministry of Health as well as other relevant NGOs and private organizations (such as such as HANDS (Health and Nutrition Development Society), Darul Sukoon, Edhi), local authorities, community leaders, and health and social care managers who were known to have worked with older adults based in Karachi and Thatta, were invited as participants for the study. Invitations were sent via email and via phone by the study coordinators. The exclusion criteria for stakeholders were individuals aged beyond 45 years or under 20 years, not associated with the elderly population, and not a permanent resident of the area. The stakeholders recruited from the urban site were between 20 and 45 years old, and an equal representation of socio-economic backgrounds, and age groups was ensured for the final study cohorts.

### Participant recruitment

The call for recruitment was advertised on September 25th, 2021, for both urban (Karachi) and rural (Thatta) study centers. A study coordinator visited the rural sites and facilitated the recruitment of participants residing within a 5–10 km radius of the chosen workshop sites from Thatta. Recruitment continued from September 26th, 2021, to September 30th, 2021, and workshops were organised on October 1st and 6th, 2021, in Thatta. Another study coordinator helped recruit participants from Karachi from October 7th, 2021, to October 12th, 2021. Workshops in Karachi were organised on October 13th and 14th, 2021. The study coordinators screened all interested participants from both sites according to the inclusion and exclusion criteria to recruit eligible participants.

### Ethics and consent

Informed written consent was acquired from the participants by the study coordinators. The participants were briefed about the study’s objectives and methods. They were oriented with the data collection procedure and were requested to sign two copies of an informed consent form, one to keep with themselves and the other to provide to the study team. Individuals who were unable to give informed consent were not included in the study. There were no specific risks to the participants in this study apart from the risk of COVID-19 transmission. Procedural adjustments were made to ensure that the end-to-end participant experience was contactless and safe. Confidentiality and privacy were maintained throughout. Approval was obtained from the AKU Departmental and Institutional Review Board (2021-6147-17944).

### Workshop design and conduct

The workshop protocol was based upon a methodology previously used by the same study team for similar workshops in Rwanda and South Africa [18,19]. Workshops were designed using the Nominal Group Technique. This technique is a structured procedure which is used to identify priorities via rapid consensus and also provides a means by which all participant voices can be heard [20,21]. The medium of instruction for the workshop was Urdu. However, translators fluent in local languages (Sindhi) were also present in the rural area (Thatta) to ensure that no meaning was lost during the discussions. Workshops (plenary and round table discussions) were also recorded in case of need for later clarity and to cross-check the results of in-person voting. The discussions were held separately for older adults and stakeholders and both study sites. While separation by gender was necessary in the rural site due to cultural considerations, such was not the case in the urban site. After summarizing the purpose and conduct of the workshops in plenary, participants were asked the first question relating to the definition of ageing, through the questions ‘How would you describe ageing?’ or ‘What do you consider ageing to be?’. This aimed to solicit the participants’ different perceptions of ageing, set the scene for the meeting, and encourage discussion. Participants were then divided into groups of a maximum of five and asked to discuss the following topics in depth. 1. What do you think is important for the ageing population to live a healthy and active life? (priorities for older people); 2. What are the main obstacles older people face that prevent them from living a healthy and active life (barriers or problems faced by elderly people)? and 3. What are the services (if any) that enable older people to live healthy and active lives? (Services and enablers available for elderly people). Each roundtable group was moderated by a facilitator who clarified the questions and guided the discussions on the topic when necessary.

During the roundtable discussions, the facilitators recorded the key points from the responses of the participants on a flipchart. After the in-depth discussion on each topic was concluded on each roundtable, each group was asked to choose their top five responses to each question. The top ranked priorities from each group were then presented in plenary to all the workshop participants, giving the whole group an opportunity to discuss why the responses were chosen and their order in the priority list. After removal of duplicates, all workshop participants were asked to vote on each response. Several rounds of voting were conducted until there was consensus on the ranking of the responses. The number of votes for each response was recorded using flip charts, and the top five priorities for each topic discussed were highlighted.

Data obtained for each group of older adult and stakeholder participants were presented as priority ranking lists consisting of single-line concise answers representing the full response provided by the participants. Researchers from Aga Khan University facilitated all activities. The ranked responses were subsequently transcribed into English.

### Geriatric depression scale (GDS) questionnaire

Study coordinators attended a two-day training program to assess mental being using the Geriatric Depression Scale (GDS). Scores of 0–4 were considered normal, 5–8 indicate mild depression, 9–11 indicated moderate depression, and 12–15 indicated severe depression, as per previous studies. Content validation was done for this purpose by translating the scale in Urdu, back translating it into English, and checking for consistency with the help of experts. While the scale itself has not been validated in the Pakistani elderly population, GDS has been used by three previous studies on this population in Karachi [22–24].

### Analyses

The final list of responses ranked according to priority were noted as the main data output of the workshops. Responses that acquired an equal number of votes were given were given the same rank level in the priority list. The data were then sorted according to location of participants i.e., priorities of inhabitants of the rural area versus the priorities of the inhabitants of the urban area, and type of participant group i.e., priorities of the elderly participants versus priorities of the stakeholders, regardless of location. Furthermore, the research team categorized the top 5 ranked responses using a four-level socio-ecological model [25], which is a framework promoted by the World Health Organisation and helps to determine how different factors interact to influence wellbeing: 1. Individual—pertaining to the internal attributes of individuals, for example, their demographics or health; 2. Relationship–pertaining to relationships proximate to the individual, for example with their family and friends; 3. Environment–being the surroundings in which person lives, including its organisations, services, and structures; and 4. Society–pertaining to the culture, social, and policy (including health, legal, and economic) milieu in which the person lives [26]. Each response was categorized into one of these levels where possible, although more could be used if a response was potentially represented by more than one level of the socio-ecological framework.

We tended towards a deterministic approach, for example, for a person to achieve their needs there needed to be provision of externalities to enable that [27]. Hence, for example, an individual’s ability to eat or exercise was considered primarily a factor of the environmental or societal provision of enablers [28]. We categorized things that are experienced by the individual, such as their own health, or feelings of loneliness for example, as ‘individual’. Financial support from family or friends was considered a relationship level response, however support in the form of discounted medical expenses was mapped on to a societal level. The first 30% of responses were categorised by CG & JD, together; the subsequent 10% of responses were categorised by each separately until 100% agreement had been achieved; after-which the remaining responses were categorised by JD. Frequency and percentages were also reported and compared for additional variables between study sites.

## Results

Workshops with stakeholders included 24 caregivers from Karachi (out of 35 invited) and 26 caregivers from Thatta (out of 30 invited) from various professional backgrounds, including community leaders and caretakers. Workshops with older people included 24 in Karachi (out of 35 invited) and 26 in Thatta (out of 30 invited). The average age of the older participants was 67.3 and 67.2 years in Karachi and Thatta, respectively (see S1 Table). In the Karachi workshop for the older adults, there were 66.7% females (16) and 33.3% (8) males. In Thatta, there were 5 females and 21 males. The elderly participants at the urban site were retired and hailed from a working-class background. Moreover, they all had received formal education up to an intermediate or undergraduate level. Most of the older adult participants recruited at the rural site hailed from the working labour class, farmers, and field workers and were either entirely uneducated or without any recorded formal education. Despite efforts to ensure equal gender representation, there was a significant difference in the number of males and females in the study group from Thatta.

**Fig 1:**
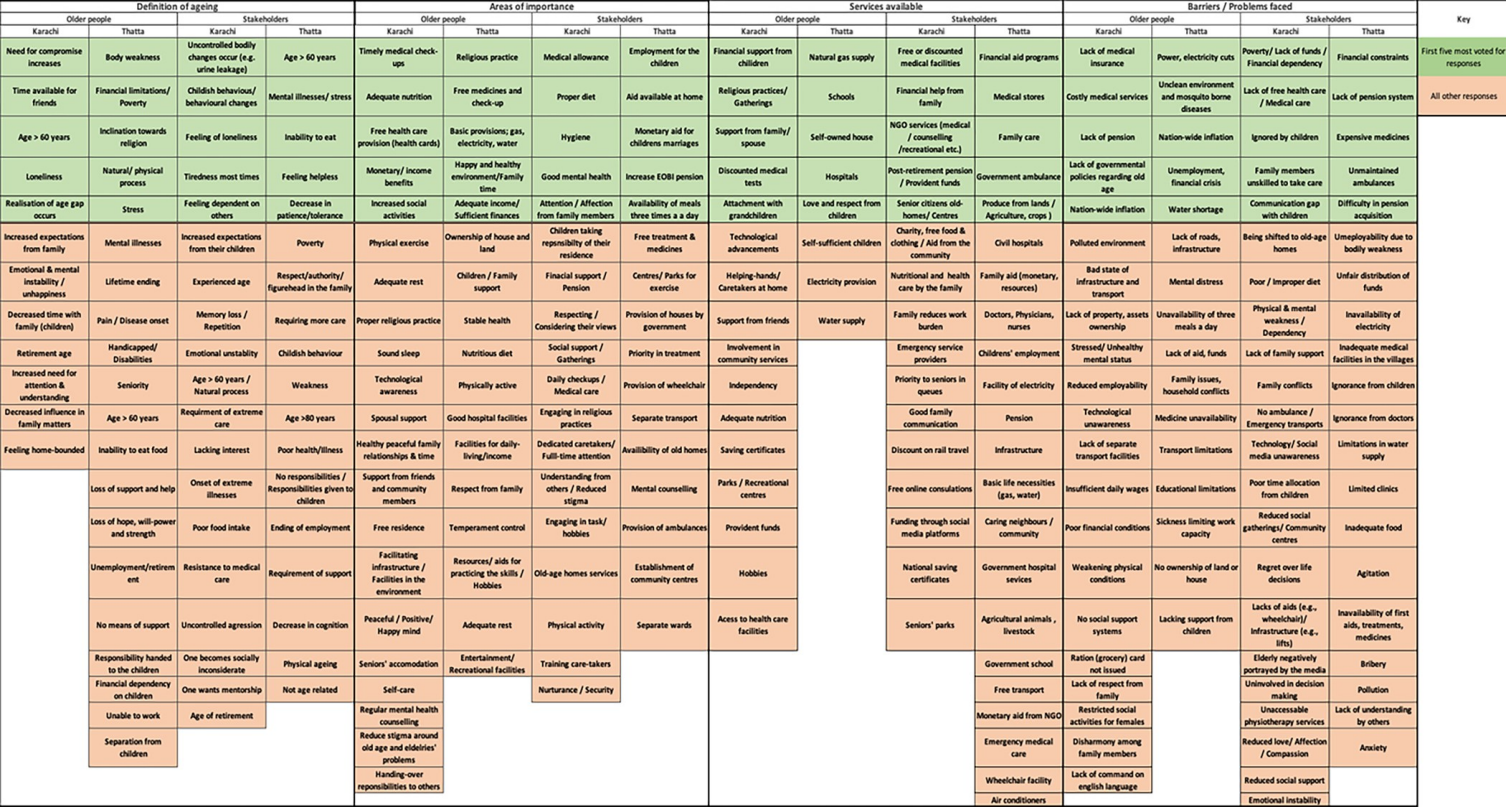
Ranked responses from the workshop participants.

For each of the group workshops, the complete list of responses to each question is shown in Fig 1.

### Contrasting the priorities of the respondent groups for healthy ageing

**Fig 2:**
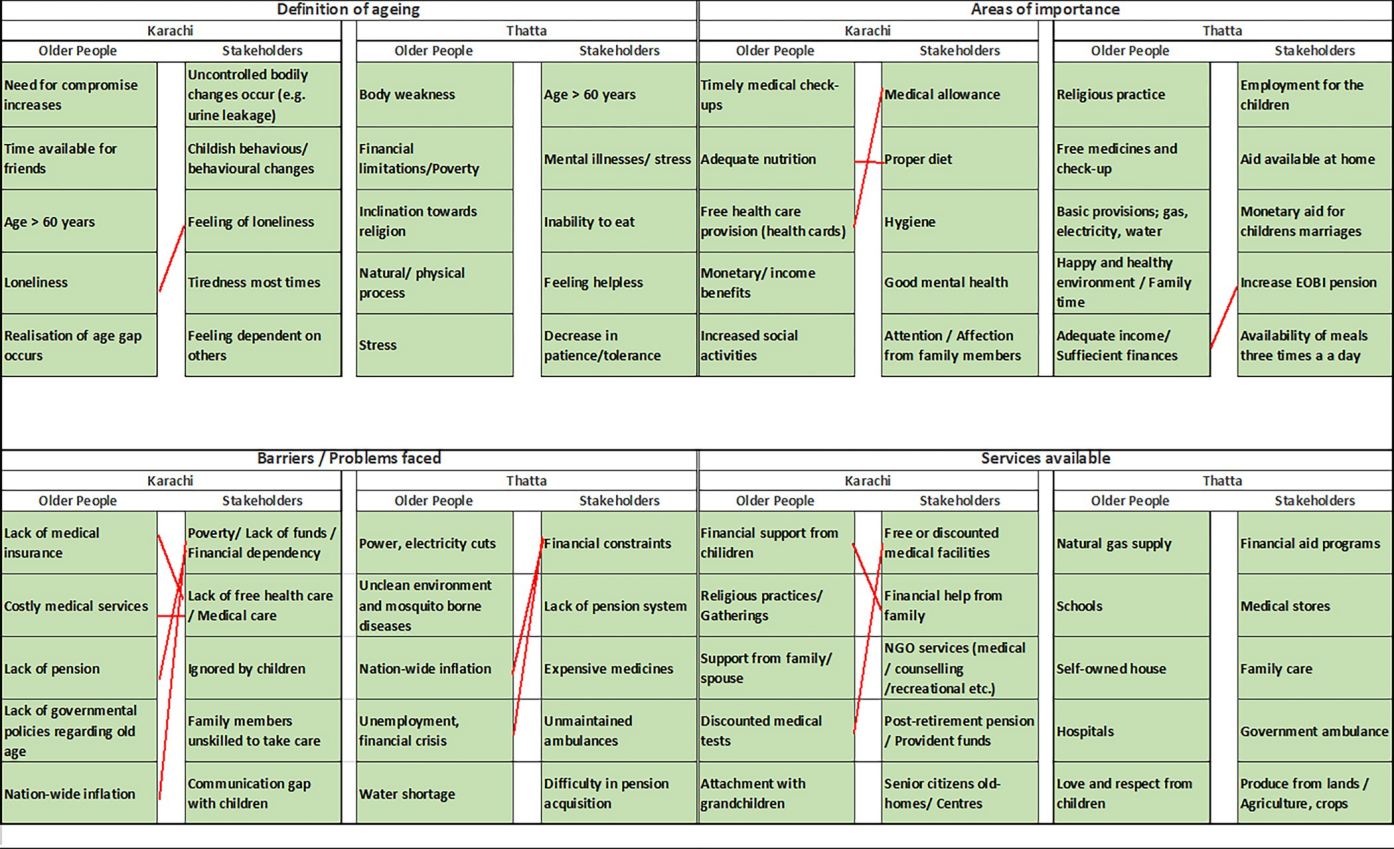
Top five ranked responses to each question. Red connecting lines indicate agreement between stakeholders and older people from each geographical area.

When comparing the top five prioritized responses of stakeholders with older people from the same geographical area, it was observed that were few areas of agreement between the two groups of respondents; 12 common responses out of the total 80 responses (Fig 2). With regard to the definition of ageing, the association of ageing with feelings of loneliness was common to both older adult and stakeholder groups in the urban area, while the two groups in rural Thatta found no common ground on the topic. With regard to areas of importance, the elderly and stakeholder groups from Karachi both prioritized good nutrition and adequate healthcare facilities as an area of importance. On the other hand, respondent groups from Thatta agreed only with the need for adequate income/ sufficient finances as a requirement for healthy ageing. Both the respondent groups from Karachi highlighted multiple issues under the theme of health, finances and government policies as barriers faced by elderly people. In contrast, the respondent groups from Thatta agreed only on the lack of finances as the main barrier to healthy ageing. According to the groups from Karachi the services available to the elderly were related to domestic financial support and discounted medical services to facilitate older people living healthy and active lives. However, there was no congruence between responses from the groups from Thatta with regard to services available to older people (Fig 2).

### Differences in priorities between geographical areas

**Fig 3:**
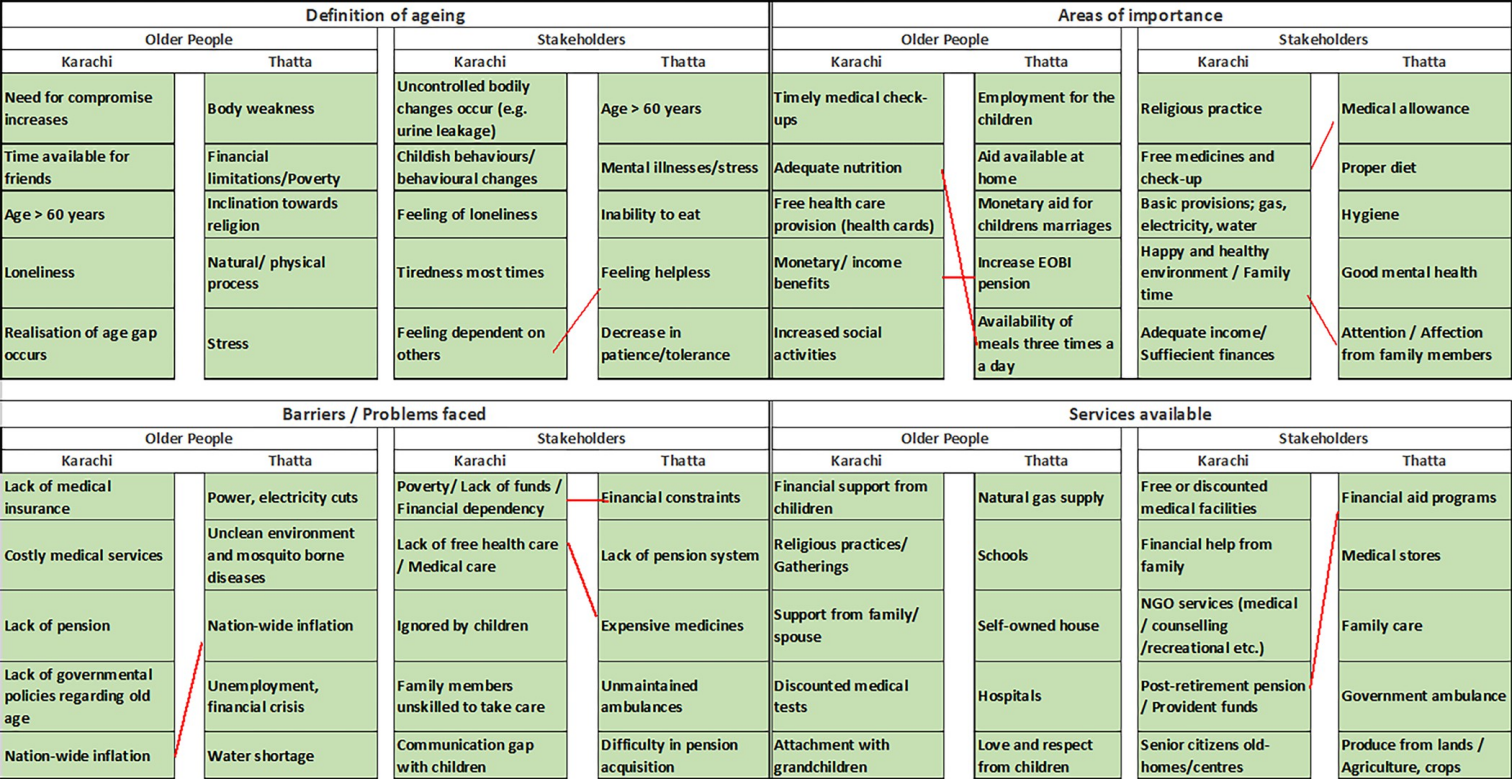
Top five ranked responses to each question. Red connecting lines indicate agreement within geographical area between each study group (older people and stakeholders).

The results showed marked differences between people from the same identity groups (either older people or stakeholders) by geography, with only 9 common responses out of the total 80 responses (Fig 3). For the definition of ageing, the most prioritized responses by the older people in Karachi were not ranked accordingly by older people from Thatta. Similarly, the stakeholders from the two geographically different regions did not share common ground on any of the definitions on ageing except for ‘feelings of dependency’. With respect to prioritized areas of importance; nutrition and finances, were the only responses shared between the older people from Thatta and Karachi. The stakeholders from both the regions ranked the importance of medical aid and emotional support from family members. The groups from both geographical regions considered lack of financial support and expenses as the main barrier towards healthy ageing, additionally the stakeholders prioritized the lack of affordable medical aid as the major problem encountered by the ageing population. The services available according to older people in Karachi did not overlap with any services voted for by older people of Thatta. The provision of post-retirement funds was the only service available to older adults, according to the stakeholders from both regions.

See S1 and S2 Figs for detailed maps exploring the linkage between responses and prioritized responses across participant groups and geographical areas.

### Socio-ecological model

**Fig 4:**
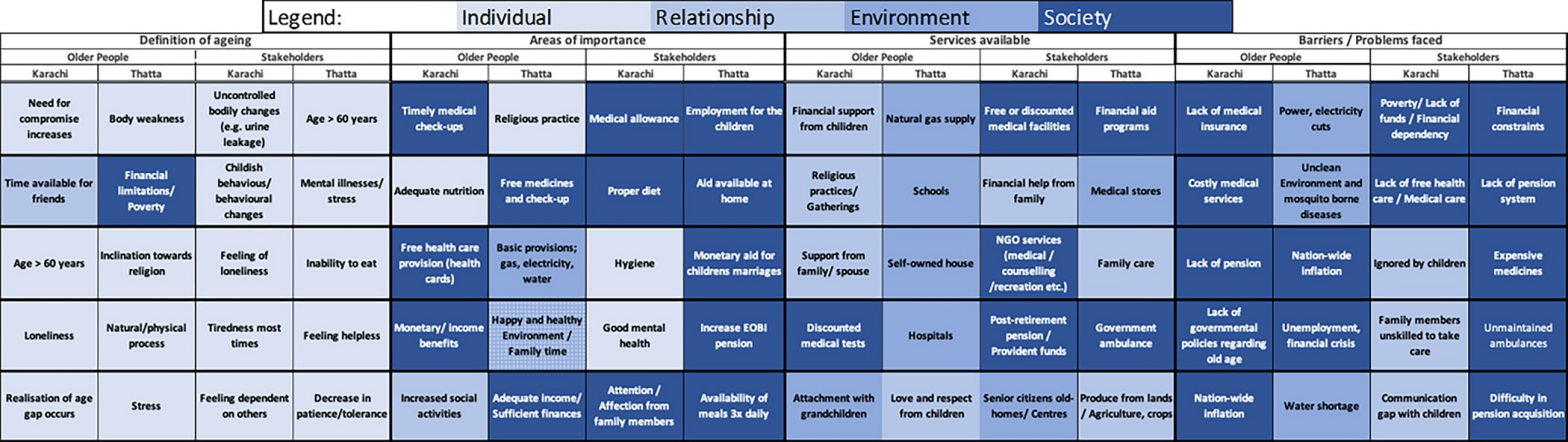
Top five ranked responses for stakeholders and older people and their related socio-ecological framework category.

The categorization of the responses for each of the discussion questions using the socio-ecological model, showed that the societal factors were prioritized most frequently (32 responses), followed by individual (27), relationship (15), and environment (14) (Fig 4). When considering the first five ranked responses to each question, different categories of the socio-ecological framework were relevant, depending on the type of respondent and their area, although there were some similarities.

For the definition of ageing, most responses were categorized as individual, regardless of geographical area or type of study population. Areas of importance for older people in Karachi were mostly categorized as societal whereas for stakeholders, most responses mapped onto individual and societal levels. For older people in Thatta, areas of importance covered all 4 levels with the highest ranked responses being classified as societal. In contrast, the responses of the stakeholders from Thatta were mainly societal level.

For barriers to healthy ageing, the older people from Karachi provided responses that were mostly societal, while stakeholder responses were categorized at the relationship level. In Thatta, responses of the older people to the barriers faced, related to the environmental level, while all stakeholders prioritized factors classified only at the societal level.

For the services available, the priorities of the older people in Karachi were mostly at the relationship level, whereas the Karachi stakeholders’ priorities were more focused on the societal level. In Thatta, responses for older people were mainly categorized as environmental and differed from the rural area stakeholder responses which were categorized equally as environmental and societal.

### Mental health assessment in older adults

Overall, 24 (48%) participants had a geriatric depression score indicating mild depression and 19 (38%) had a score indicating moderate or severe depression. The occurrence of moderate and severe depression was approximately 5 times higher in Thatta than in Karachi (62% and 13%, respectively) (see S1 Table).

## Discussion

There are limited data reporting the needs and priorities associated with an increasing ageing population in Pakistan. Moreover, previous studies exploring older populations in Pakistan have remained centric to a single study population and have not covered the scope of socioeconomic and demographic variation and its impacts on healthy ageing. This is the first study in a lower middle-income country that aimed to identify and compare the views of older people and stakeholders on their definition of ageing and areas of importance, barriers, and resources needed to enable older people to live healthy active lives. Moreover, this study compares older adults and stakeholder groups across different geographical settings.

Residents of Thatta in comparison to residents of Karachi are severely deficient of basic health-care facilities and provisions [17] The data show significant differences between participants from Thatta and Karachi, with our analysis suggesting that the responses to questions about definitions, areas of importance, experienced barriers, and postulated enablers of healthy ageing, are influenced by the financial and physical hardships experienced by older adults living in a rural compared to an urban area of Pakistan.

While older adults from the urban region included emotional changes i.e., loneliness and dependency within their top-ranked definitions of ageing, older adults from the rural area associated ageing with physical changes, and a decline in financial resources. Interestingly the responses of older people in Thatta included more physical and mental health features in their definition of ageing, than those in Karachi. These differences can be attributed to the fact that the population from the rural area were primarily men, fieldworkers and laborers, whose main source of income was through farming and labor-work, therefore a decline in their physical fitness with age reduced their ability to cater to themselves and their families’ financial needs. A previous study of older adults from peri-urban areas showed that the majority wanted to continue working and remain independent of their children’s earnings [29]. According to a recent study, most elderly adults above the age of 60 years want to remain active and be useful contributors to society. However, their old age limits their employability options. Moreover, pensionable elderly adults have reported that their pensions are not adjusted according to rising inflation patterns. Consequently, they find themselves financially handicapped and unable to access mandatory healthcare facilities or resources. This is consistent with another study which identified lack of support systems, aloof behavior of others, poverty and feelings of loneliness as drivers for elderly people to move to shelter homes [15]. On the other hand, elderly people from Karachi, were mainly retired pensionable individuals for whom major life-changes attributable to ageing were feelings of disconnect with family members, emotional decline and reduced importance in the household. The inclusion of domestic changes associated with ageing like ‘household importance’, and ‘reduced family attention’ among the responses may be linked to the fact that the urban group (unlike the rural group), was equally characterized by females. Unlike elderly people in Thatta, loneliness was mentioned only by the Karachi inhabitants, thus we inferred that the challenges of older people living in an urban area may be as a result of them living independently of younger working family members. Moreover, this reflects the recent transition of the Pakistani family system to nuclear families at a higher rate of progression in urban than in rural areas of the country [30,31]. The responses of the elderly groups, i.e., loss of emotional and financial support, uncontrolled changes, compromise, home-bound, inability to earn etc., may have arisen as a result of having low perceived control, reduced social support, and reduced self-esteem, that are commonly associated with ageing [32].

When questioned about areas of importance for healthy ageing, medical resources were the most frequent responses in the elderly and stakeholder respondent groups from Karachi and the aged of Thatta. However, this was not a top priority for the stakeholders from Thatta, rather they pressed on the need for employment and monetary funds for the children of elderly people. This seems at odds with the importance ascribed by older people to the maintenance of functional ability through improved health care, which is crucial to healthy ageing [33]. Other studies have also highlighted that major limitations in geriatric care are a result of the lack of awareness, poor support; carelessness and negative attitude of the stakeholders towards elderly population in Pakistan [34]. Caregivers and older adults from Sri Lanka also highlighted limited referral facilities, infrastructure, financial support, lack of trained personnel and geriatric services as barriers to quality of care in ageing [35]. Notably, the stakeholders from Karachi highlighted the importance of both mental and physical health as well as the need for financial stability and emotional wellbeing, indicating that the city’s youth and key individuals for shaping geriatric care acknowledged all the essential requirements for older adults. It was also noted that religion was repeatedly ranked as ‘part of ageing’ and an ‘important need’ for Thatta’s elderly people, while this was not the priority of elderly people in Karachi. Moreover, unlike the Thatta population, the elderly from Karachi prioritized social activities. This difference in the priorities results from the socioeconomic and cultural variation between the two regions which has determined the types of lives elderly people had led in their youth, and consequently their areas of importance for healthy ageing. This finding is similar to the observations of a study conducted in Iran, in which the participants responses suggested a marked effect of cultural and socio-economic status in the past, living in urban and rural areas, beliefs and attitudes, and financial capacity as the major the cultural and socio-economic factors influencing their opinions and behavior with aging [36]. Overall, the results showed financial constraints featured more frequently in the list of areas of importance for Thatta than Karachi, indicating the scarcity of resource and essential provisions in the rural area. Another study on urban elderly dwellers in China underscored the complexities of accessing care and lack of information as obstacles to care seeking [37].

Analysing the responses to ‘services available’, it was inferred that the Thatta population, regardless of age group, mainly identified provision of basic infrastructure, i.e., schools, natural gas, self-owned houses, crop produce, hospitals, rather than services specific to the needs of elderly people. On the other hand, the inhabitants of Karachi, regardless of age, considered benefits additional to basic services, such as elderly discounts, old homes, elderly-friendly recreational areas, post-retirement schemes and emotional support as the most important services. This discrepancy in the resource type level highlighted by the two geographically different groups reiterated the lack of resources and systems for older people in rural areas and highlighted severe inequality in the two populations’ living standards.

Moreover, it was observed that while the stakeholders of Thatta frequently mentioned financial and medical support as services available to elderly people, the older adult groups did not. Therefore, it may be inferred that the stakeholders from the rural areas are somewhat disconnected from the actual experiences of elderly people in terms of ability to access and receive support. This highlights an important issue prevailing in the rural areas of Pakistan; key opinion leaders and those individuals actively involved in the development sector perceive that adequate systems are in place for everyone.

In terms of barriers to healthy ageing, the inhabitants of Thatta highlighted fundamental infrastructure-related issues such as water supply and cleanliness. The stakeholders from Thatta focused only on financial limitations. In contrast, the inhabitants of Karachi prioritized problems relating to old-age needs, such as pensions and family support. Moreover, stakeholders from Karachi mentioned the lack of emotional support from family as one of the issues faced by elderly people. Also, it was noted that while the stakeholders from Karachi considered emotional support to be a significant barrier for elderly people, the elderly themselves did not consider this issue among their priorities. Unlike other topics discussed, the stakeholders and elderly from Thatta both ranked financial limitations as a barrier, which showed that the stakeholders acknowledged this problem. However, the rural stakeholders did not prioritize infrastructure-related issues, suggesting that they did not realize the severity of resource limitations. The socio-ecological mapping showed that the majority of the areas of importance and barriers related to ageing were categorized as societal, while a majority of the responses for the services available were mapped into environmental and relationship levels. This disconnect potentially highlights the lack of actioned policy and enabling services which could facilitate health and well-being in older age regardless of whether in an urban or a rural area. That said, policy for older people does exist, some of which has been put into action. In 1976, the Old Age Benefit Scheme 1976 [38] was launched to provide financial support to older individuals and in 2014, Pakistan passed the Senior Citizens Act, which laid the policy proposals and implementation of senior citizen safeguarding under the Senior Citizen Welfare Council which aims to improve access to financial and healthcare resources for older adults [8]. As a result of these policies, senior citizen homes have been established, as well as a senior citizens card that provides free access to public spaces, separate access to medical treatment, and financial support for those in need [39]. Alongside the Senior Citizens Act, many provinces established individual welfare councils and programs to protect the older community in the smaller provinces. However, despite these initiatives, provisions such as shelter/ housing, income support program, social security, free medical care, and financial stability are not readily available [40,41], reflecting the barriers reported in our study. Additionally, only 2.3% of the population older than the statutory pensionable age in Pakistan actually receives an old-age pension (contributory, non-contributory, or both) [8,42]. The private sector of Pakistan is working towards providing better facilities to the elderly alongside non-governmental organizations such as the Aga Khan foundation and Edhi Trust. However, these have limited coverage and localized effects [34]. This is reflected in the finding that while NGOs were mentioned as one of the resources available by elderly people, income and finances were seen as top ranked areas of importance and barriers for older people regardless of location. Despite the likely greater wealth of people living in Karachi compared with Thatta, which is one of the lowest performing districts in terms of socio-economic development in Pakistan, financial limitation was a problem highlighted by both groups of elderly people [43]. These findings are supported by a recent study that found older adults in care homes in Pakistan reported poverty as a major barrier to life satisfaction, followed by negligence by children [14].

The prioritized responses of older individuals and the stakeholders also contrasted substantially, both when considering the responses as captured and when categorizing these using the socio-ecological model. This may be explained by the differences in lenses of respondents, with older people responding based on their lived experience in contrast to stakeholders who may have the experience of caring for someone who is older in a personal capacity in addition to responding to questions in a professional capacity. A striking example of the difference in responses between stakeholders and older people was highlighted when considering the definition of aging, where stakeholders were more likely to define Individual characteristics, whereas older people included more external Society or Relationship factors, suggesting that the lived experience of ageing is very different to that observed. Like many developing countries, old age in Pakistan is set at 60 years and older [4,15] similar to high income countries, despite there being substantial differences in current life expectancy. Being an older individual, on the other hand, is not simply a function of chronological age and the diseases and physiological changes which are associated with this process [44]. In Pakistan, the word frailty (Kamzori) is often used to characterize old age (Burhaapa), and old age is also commonly defined by the role of eldership [4,15], but this seems in contrast to the individual socio-ecological categorization given to the definition, especially by stakeholders. It is reported elsewhere that many older individuals verbalise the ageing process and declining culture of reverence as one of the leading causes of distress and anxiety [45].

When considering areas of importance, there was also a divergence in priorities between stakeholders and older people. Stakeholders were focused on factors that could be provided by society, and whilst societal factors featured in older peoples’ priorities, they included a breadth of other categories of areas of importance, again reflecting differences in the experiences of healthy ageing and the associated barriers and enablers.

The differences in prioritized responses between urban and rural participants as well as the differences between old people and their caretakers is important to recognize. This suggests that a one-size-fits-all approach to improving health and wellbeing in older populations will not work. It also suggests that the views of stakeholders, who may be more often listened to in discussion of needs to inform planning and policy, especially if they are from an urban area, may not adequately reflect the needs of the people that they supposedly represent. Consulting the population relevant to developing services, perhaps using community participatory methods would likely bridge this gap. A similar divergence in priorities between geographical areas and stakeholder groups was found in another study in Rwanda [46].

Finally, we found that respondents from Thatta reported a higher geriatric depression score, i.e., moderate to severe depression, in contrast to those from Karachi, who reported a lower geriatric score, i.e., no to moderate depression. The results of our study are consistent with previous studies, noting a higher prevalence of depression in rural areas as compared to their urban counterparts [47,48]. According to a study on the factors strongly associated with depression in elderly people of rural India, age above 70 years, financial dependency and having one or more severe comorbidities were identified [48]. These findings also concur with findings from the workshop where stress, mental illness, and feeling helpless were responses in the definition of ageing given by both older people and stakeholders in Thatta. Respondents based in rural settings such as Thatta are more likely to present with increased severity of depression due to a variety of possible reasons. These may include inaccessibility to adequate healthcare services, financial constraints, family problems, deteriorating health, and more [18,48]. Previous studies also note some of the critical determinants contributing to higher depression in rural areas, namely economic disparity, familial disputes, regressive cultural norms, unemployment, and health-related concerns, including poor access to healthcare [17,47,49]. Moreover, it is important to acknowledge the role of a lack of awareness regarding mental health in rural settings. Urban settings are generally more well-informed about concepts like mental wellbeing in contrast to rural settings where mental health wellbeing is generally underestimated. Individuals may present with the signs and symptoms characterizing a mental health condition; however, they may not acknowledge or recognize it owing to a lack of mental health literacy [50].

This study is not without limitations. Even though the comparison of two cities in Pakistan in this study focussed attention on a number of important commonalities and differences, and participant responses appeared to reflect the current inter-regional disparity regarding healthcare, financial, infrastructure etc., services/provisions for the elderly across Pakistan, they may not fully represent the whole ageing population of this country. As time and resources limited data collection to one urban and one rural area, these results may not represent all rural and urban areas in Pakistan, where approximately 14 million adults aged over 60 years currently reside. The under-representation of older females in Thatta, mainly due to cultural norms of the rural areas is another limiting factor. We also acknowledge the limitation of using small group methodologies such as the Nominal Group Technique; while the most suitable technique to address our research questions, it restricted numbers of participants and workshops. However, we would like to emphasise that this was an exploratory study, which was intended as a springboard to stimulate more engagement and dialogue between older adults and the key stakeholder groups who care for them. Owing to the study’s exploratory nature, we aimed to acquire an in-depth understanding of the concepts around ageing in a setting where such concepts had not been explored in the past. Despite the limitations, these workshops provided opportunities to grow a small body of literature on healthy ageing needs and priorities in LMICs.

### Conclusion

This study sheds light on the crucial need for tailored interventions that address the diverse concerns of older adults and their caretakers, catering specifically to the unique challenges faced in both urban and rural settings. Achieving healthy ageing requires a comprehensive and inclusive approach, encompassing the perspectives of all stakeholders, including non-governmental organizations (NGOs), public and private institutions, the older adult population themselves, and their caretakers. Our findings serve as a valuable resource for researchers and policymakers aiming to raise awareness amongst elderly people and various stakeholders across Pakistan about the importance of healthy ageing and the associated challenges, as well as inform the development of comprehensive strategies and policies, coupled with necessary infrastructural improvements, to facilitate and empower healthy ageing in low- and middle-income countries (LMICs) like Pakistan. By incorporating the varied perspectives of diverse stakeholders and older adults residing in different geographical locations, this study paves the way for the creation of effective and context-specific solutions that truly prioritize the well-being of the ageing population throughout Pakistan.

### Policy implications

In the context of ageing in urban and rural Pakistan, a roadmap for effective action emerges. Prioritizing equitable healthcare access is paramount, with a special focus on underserved rural regions. Investment in healthcare infrastructure, the introduction of mobile healthcare units, and specialized health insurance schemes for the elderly are essential steps. Additionally, creating age-friendly urban environments, with improved public facilities catering to the unique needs of seniors, should be a central policy concern. Bolstering social security, enabling elderly workforce participation, enhancing mental health services, and implementing protective measures against elder abuse are vital for improving senior citizens’ quality of life.

In terms of research, comprehensive longitudinal studies are recommended to gain insights into the evolving needs of ageing populations. Exploring the role of technology in enhancing elderly lives, examining inter-generational dynamics, understanding cultural factors’ influence on ageing, and evaluating community-based support systems are key research avenues. Assessing the economic contributions of the elderly and the impact of health education initiatives on preventive healthcare and healthier lifestyles should also be central themes. These measures, when applied with cultural sensitivity and transparency, can bridge the gap between policy intentions and on-ground impact.

Like many countries, Pakistan grapples with the challenge of translating well-crafted policies into effective action. While policies on healthcare, education, and social welfare exist, their practical impact often falls short due to bureaucratic obstacles, resource limitations, and inadequate monitoring. For example, healthcare policies may struggle with insufficient infrastructure and skilled personnel, while education policies may not ensure access to quality teaching and resources. This gap between policy formulation and implementation underscores the importance of investing in essential resources, building institutional capacity, and establishing robust monitoring systems. Fostering a culture of accountability and transparency is equally crucial to ensuring that policies effectively benefit Pakistan’s people and advance socio-economic progress.

## Supporting information

S1 Fig Detailed map of responses and similarities between stakeholders and older people in both rural and urban areas. The responses from stakeholders are displayed as green colored text while the responses from the older adults are displayed as red colored text. The frequency of the responses are denoted by the weight of boldness of the text, and the highlight shows that the response was common between stakeholders and older adults. The responses obtained from a specific study are aligned accordingly i.e., the responses from Thatta fall towards the right of the heading, while the responses from Karachi fall towards the left of the heading. The black connectors show the relationship between the response to the four questions. The colored connectors describe the interrelatedness between the responses to the different questions.
(PDF)

S2 Fig Detailed map of prioritized responses and similarities between stakeholders and older people in both rural and urban areas. The colored backgrounds are used to categorize the response based on study site differences and similarities. The Stakeholder responses are displayed as green text while the older adult responses are displayed as red colored text. The highlight shows that the response was common between the stakeholders and the older adults. The black connectors show the relationship between the four questions. The colored connectors describe the interrelatedness between the responses to the different questions.
(PDF)

S1 Table Physical and mental health characteristics of the older participants. (DOCX)

S1 File (DOCX)
