# Supplementary material for: Exploring the priorities of ageing populations in Pakistan, comparing views of older people in Karachi City and Thatta
Source: PLoS One. 2024 Jul 5;19(7):e0304474. doi: 10.1371/journal.pone.0304474 (PMC11226073; doi:10.1371/journal.pone.0304474)
Supplement: S1 Table — (DOCX) [file pone.0304474.s003.docx]

Supplementary Table 1: Physical and mental health characteristics of the older participants

| **VARIABLES** | **N=50 mean (sd)** | **THATTA mean (sd)** | **KARACHI mean (sd)** |
| --- | --- | --- | --- |
| **Age in years^** | 67.3 (5.4) | 67.2 (5.8) | 67.3 (5.0) |
| **60 – 64 n (%)** | 17 (34) | 10 (38.5) | 7 (29) |
| **65 – 69 n (%)** | 17 (34) | 6 (23) | 11 (46) |
| **70 + n (%)** | 16 (16) | 10 (38.5) | 6 (25) |
| **Geriatric Depression Score** | 7.9 (3.1) | 9.6 (2.4) | 5.9 (2.4) |
| **No Depression** | 7 (14) | 0 (0) | 7 (29.2) |
| **Mild Depression** | 24 (48) | 10 (38) | 14 (58.3) |
| **Moderate or severe depression** | 19 (38) | 16 (62) | 3 (13) |
