# Supplementary material for: Exploring the priorities of ageing populations in Pakistan, comparing views of older people in Karachi City and Thatta
Source: PLoS One. 2024 Jul 5;19(7):e0304474. doi: 10.1371/journal.pone.0304474 (PMC11226073; doi:10.1371/journal.pone.0304474)
Supplement: S1 Fig — The responses from stakeholders are displayed as green colored text while the responses from the older adults are displayed as red colored text. The frequency of the responses are denoted by the weight of boldness of the text, and the highlight shows that the response was common between stakeholders and older adults. The responses obtained from a specific study are aligned accordingly i.e., the responses from Thatta fall towards the right of the heading, while the responses from Karachi fall towards the left of the heading. The black connectors show the relationship between the response to the four questions. The colored connectors describe the interrelatedness between the responses to the different questions. (PDF) [file pone.0304474.s001.pdf]

Supplementary Figure 1: Detailed map of responses and similarities between stakeholders and older people in both rural and urban areas

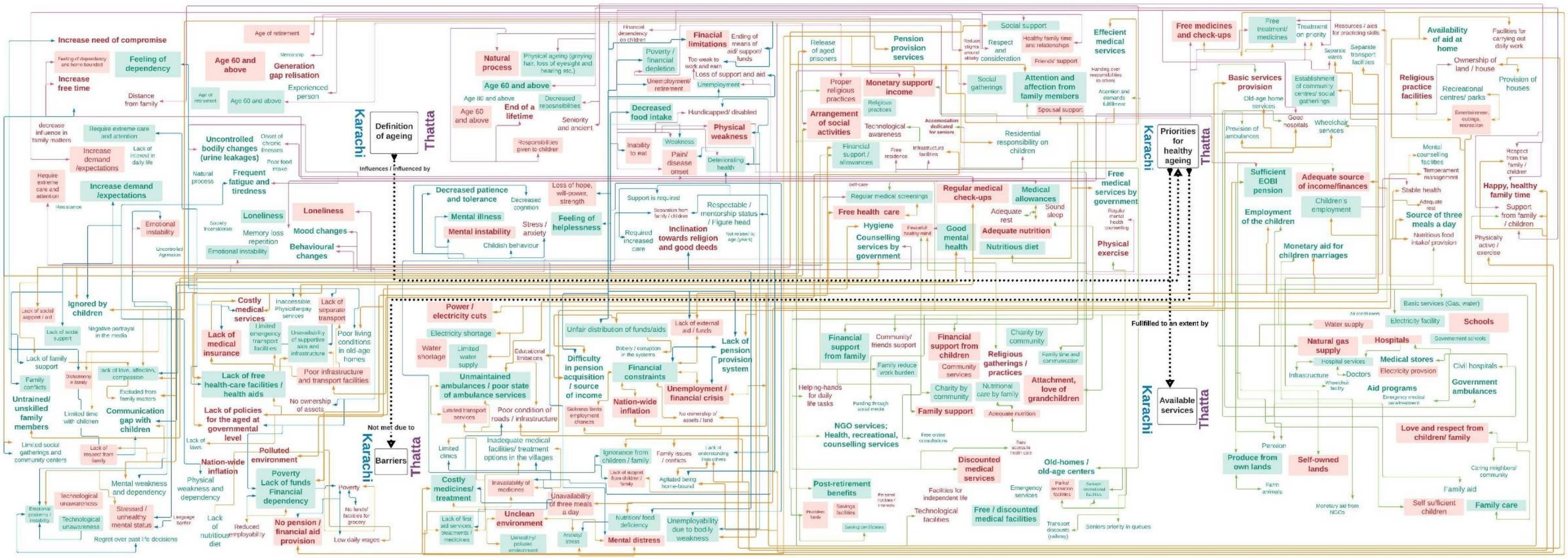

The responses from stakeholders are displayed as green colored text while the responses from the older adults are displayed as red colored text. The frequency of the responses are denoted by the weight of boldness of the text, and the highlight shows that the response was common between stakeholders and older adults. The responses obtained from a specific study are aligned accordingly i.e., the responses from Thatta fall towards the right of the heading, while the responses from Karachi fall towards the left of the heading. The black connectors show the relationship between the response to the four questions. The colored connectors describe the interrelatedness between the responses to the different questions.
