# Supplementary material for: Exploring the priorities of ageing populations in Pakistan, comparing views of older people in Karachi City and Thatta
Source: PLoS One. 2024 Jul 5;19(7):e0304474. doi: 10.1371/journal.pone.0304474 (PMC11226073; doi:10.1371/journal.pone.0304474)
Supplement: S2 Fig — The colored backgrounds are used to categorize the response based on study site differences and similarities. The Stakeholder responses are displayed as green text while the older adult responses are displayed as red colored text. The highlight shows that the response was common between the stakeholders and the older adults. The black connectors show the relationship between the four questions. The colored connectors describe the interrelatedness between the responses to the different questions. (PDF) [file pone.0304474.s002.pdf]

Supplementary Figure 2: Detailed map of prioritized responses and similarities between stakeholders and older people in both rural and urban areas

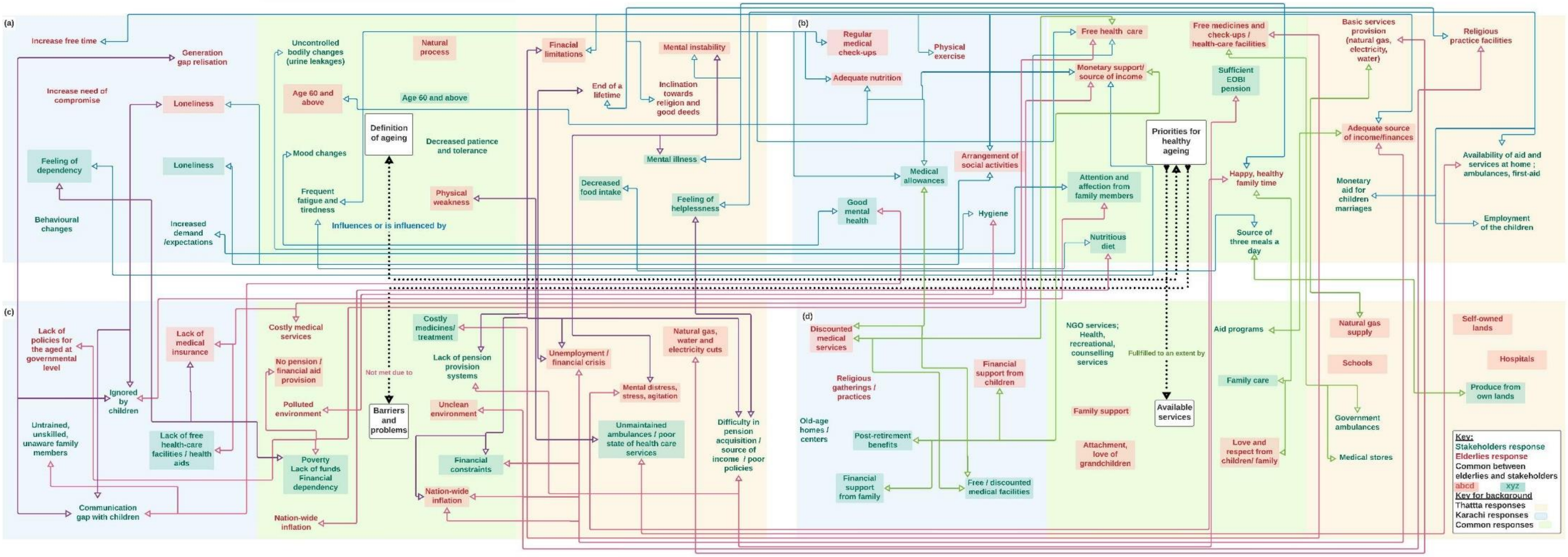

The colored backgrounds are used to categorize the response based on study site differences and similarities. The Stakeholder responses are displayed as green text while the older adult responses are displayed as red colored text. The highlight shows that the response was common between the stakeholders and the older adults. The black connectors show the relationship between the four questions. The colored connectors describe the interrelatedness between the responses to the different questions.
